# Absence of Leishmaniinae and Nosematidae in stingless bees

**DOI:** 10.1038/srep32547

**Published:** 2016-09-02

**Authors:** Patrícia Nunes-Silva, Niels Piot, Ivan Meeus, Betina Blochtein, Guy Smagghe

**Affiliations:** 1 Laboratory of Entomology, Department of Biodiversity and Ecology, Faculty of Biosciences, Pontifical Catholic University of Rio Grande do Sul, Brazil; 2 Laboratory of Agrozoology, Department of Crop Protection, Faculty of Bioscience Engineering, Ghent University, Belgium

## Abstract

Bee pollination is an indispensable component of global food production and plays a crucial role in sustainable agriculture. The worldwide decline of bee populations, including wild pollinators, poses a threat to this system. However, most studies to date are situated in temperate regions where Apini and Bombini are very abundant pollinators. Tropical and subtropical regions where stingless bees (Apidae: Meliponini) are generally very common, are often overlooked. These bees also face pressure due to deforestation and agricultural intensification as well as the growing use and spread of exotic pollinators as *Apis mellifera* and *Bombus* species. The loss or decline of this important bee tribe would have a large impact on their provided ecosystem services, in both wild and agricultural landscapes. The importance of pollinator diseases, which can contribute to decline, has not been investigated so far in this bee tribe. Here we report on the first large pathogen screening of Meliponini species in southern Brazil. Remarkably we observed that there was an absence of Leishmaniinae and Nosematidae, and a very low occurrence of *Apicystis bombi*. Our data on disease prevalence in both understudied areas and species, can greatly improve our knowledge on the distribution of pathogens among bee species.

Stingless bees (Meliponini), the Neotropical sisters of the other highly social Apini, are native to several continents, including South America, Australia, Asia and Africa and provide important pollination services on a variety of crops and wild plants^1^,^2^. As reported for bee species in temperate regions^3^,^4^, these bee species are also under pressure. Deforestation leads to habitat loss, reducing the availability of suitable nest sites as well as food resources, and agricultural intensification increases the exposure to pesticides^5^,^6^,^7^,^8^. Invasive species, particularly the intensified use of introduced honeybees along with other exotic pollinators, have an impact on the native wild bees. These introduced bees compete for food resources and are a potential host and spreader of emerging infectious diseases^7^,^9^,^10^. Several of the known honeybee pathogens have been detected in Brazilian honeybees as well as in introduced bumblebees in neighboring countries^11^,^12^. As most of these honeybee pathogens are found in a range of Apoidea species^13^,^14^, the host range of these pathogen families will probably encompasses the Meliponini which are closely related to Apini^15^. For honeybee RNA viruses, that have a broad host range^14^, this has indeed been reported^16^. The host range of the protozoan and microsporidian pathogens found in honeybees is probably more restricted. However most of these pathogens have been found in a range of Apoidea species^13^ which are more distantly related to honeybees than Meliponini (Fig. 1). Protozoan (*Crithidia* sp.) and microsporidian (*Nosema* sp.) pathogens have been suggested as an important driver of decline in native bumblebees in North and South America^17^,^18^. However the susceptibility of stingless bees to these protozoan and microsporidian pathogens, which appear to affect most bee families within the Apoidea, is still unknown. We therefore performed a large pathogen screening on six stingless bee species in the south of Brazil. We used broad range detection methods to enable the detection of possible new pathogen species. This large pathogen screening of Meliponini species contributes to the current knowledge of bee disease as one of the drivers of decline.

## Results and Discussion

To examine the prevalence of protozoan and microsporidian pathogens in Meliponini, 150 colonies of six different species, i.e. *Melipona bicolor, Plebeia droryana*, *Plebeia emerina*, *Plebeia remota*, *Plebeia saiqui, Tetragonisca fiebrigi*, were sampled as well as honeybee (*Apis mellifera*) colonies. Colonies were sampled at nine different locations in the south of Brazil. All six stingless bee species are native to Brazil and to neighboring countries except for *P. saiqui* which is only found in Brazil. The colonies were screened with broad range primers for Leishmaniinae (Kinetoplastida:Trypanosomatidae), Nosematidae (Microsporidia: Apansporoblastina) and Neogregarinorida enabling detection of all described pathogens as well as potential novel species. To our great surprise no Leishmaniinae nor Nosematidae were detected in any of the 131 stingless bee colonies at the nine different locations; in total more than 1900 stingless bee specimens were tested (Supplementary Table S1). In great contrast, we detected both Leishmaniinae and Nosematidae in honeybees. *Lotmaria passim* (Trypanosomatidae: Leishmaniinae) was found in two out of the four locations where honeybees were sampled (Fig. 2). In Cambará do Sul, 80% (n = 10) of the screened hives were positive and in Porto Alegre all honeybee hives (n = 4) were positive. Similarly, we detected the microsporidian *Nosema ceranae* (Microsporidia:Nosematidae) in honeybees in Cambará do Sul (10%) and Porto Alegre (100%). Overall we found an infection percentage of 63% and 26% in honeybees (n = 19) for *L. passim* and *N. ceranae*, respectively. For the neogregarine, *Apicystis bombi*, the prevalence data were different (Fig. 2). In the Meliponini species, *A. bombi* infections were detected in two species, i.e. *P. emerina* and *T. fiebrigi*, however this was at a very low prevalence of 3% of the stingless bee colonies (n = 131). *A. bombi* was detected in Rolante with 50% infection in *P. emerina* colonies (n = 4) and 10% infection in *T. fiebrigi* colonies (n = 10). In Herval d’Oeste, we found 7.7% infected *T. fiebrigi* colonies (Supplementary Table S1), but to our surprise, we did not find *A. bombi* in any of the honeybee hives tested (n = 19) over the 4 locations (Fig. 2).

**Figure 1:**
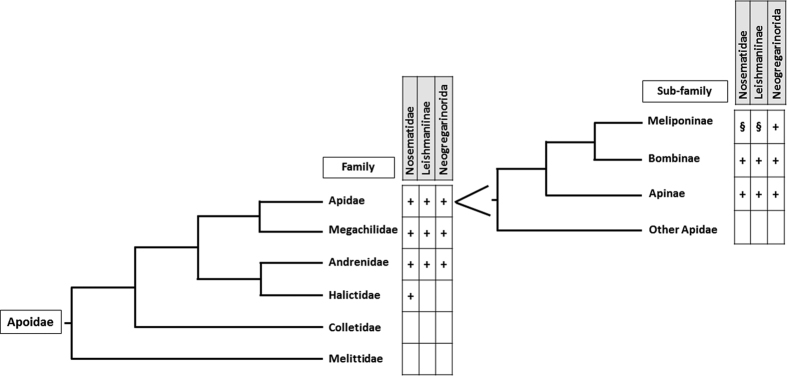
Schematic representation of phylogenetic relation in the order of the Apoidae after Hedtke *et al*.^34^ (the small family Stenotritidae was not included) and the current knowledge on pathogen presence^13^,^27^,^35^ (Nosematidae, Leishmaniinae and Neogregarinorida). ‘+’ indicates reported presence. ‘§’ indicates the expected presence yet not found in our study. Empty blocks indicate an absence of data.

This unique observation where Nosematidae and Leishmaniinae were absent in the six screened Meliponini species is very remarkable especially since we know that these pathogens are present in Brazilian honeybees^19^ and bumblebees native to South America^11^, which share flowers with stingless bees^20^,^21^. Flowers are known to be a hotspot for infection, enabling interspecies transmission of bee pathogens^22^,^23^. Moreover honeybee corbicular pollen, which has been shown to often contain protozoan and microsporidian pathogens^24^, is regularly used in meliponiculture as food source during periods when less flowers are available. The absence of these pathogens in stingless bees can therefore not be due to lack of inoculum. One hypothesis is that there is a genus barrier which prevents infection. Another one is that stingless bees could be more resistant to these pathogens because the infection success would be lowered through lifestyle, providing an extra defense against these pathogens on top of the internal immune system^25^. Unlike honeybees, stingless bees construct brood cells with a mixture of wax and propolis. Interestingly, feeding of stingless bee propolis has been shown to lower the progression of *Nosema* infections in honeybees^26^, suggesting a potential role of stingless bee propolis against *Nosema* infections. Indeed it is to be underlined that the absence of Leishmaniinae and microsporidia in the stingless bees is remarkable as we screened more than 1900 individuals in total. Both pathogen families have hosts in the Apidae, other families of the Apoidea^13^ (Fig. 1) as well as a wide array of other insect species^27^. However, so far no active replication has been shown in the Apoidea species other than Bombini and Apini.

The presence of *A. bombi* in the stingless bees could indicate that there is spillover from honeybees or bumblebees, as this pathogen is well described for both and seems to be present in neighboring countries at a quite high prevalence^11^,^28^,^29^. However no infected honeybees were found in our study. It could be possible that we missed the presence in honeybees because honeybee hives were not present in every location studied and consequently not sampled. Yet we checked honeybees at the locations where *A. bombi* was found in stingless bees and no *A. bombi* was found in these honeybees. Africanized honeybees are known for swarming which could result in the establishment of honeybee colonies in more remote regions, increasing contact with native stingless bees. Moreover *A. bombi* has been found in wild bumblebees (*B. atratus*) native to South America^11^. *A. bombi* is also present in corbicular pollen^24^ often fed to stingless bee hives.

Our sequence analysis of the ITS region of one of the infected *T. fiebrigi* hives shows a close match to European *A. bombi* (Supplementary Figure S1), however as there tends to be little variation in the ITS region of *A. bombi*^29^ one has to be careful in drawing conclusions upon the geographic origin of the pathogen, as recently shown for *Nosema bombi*^30^.

So at the end of our research, we believe that the absence of pathogens in stingless bees is not due to a lack of inoculum, however the source (i.e. the dominant spreader, as well as the geographic origin) of the inoculum remains uncertain. Our findings support the current promotion of meliponiculture and the use of domesticated Meliponini species in Brazil^31^ for pollination services, as they are effective pollinators of several crops and appear to be free of Leishmaniinae and Nosematidae pathogens. We want to stress here extra on previous cases in Europe and North America to be precautious with the translocation of ‘managed’ honeybee and stingless bee hives as these can spread pathogens to wild pollinators. Also the use of honeybee corbicular pollen is to be taken with care as this has been shown to be a possible source for pathogens^24^.

Even though Meliponini species seem to be free of most pathogens, the knowledge about the diseases or parasites of this group is still very restricted. Therefore more scientific research and careful sanitary and regulatory recommendations in the use of honeybee products in meliponiculture and transport of managed pollinators, are required for bee conservation in Brazil and other countries in the tropical and subtropical region.

## Methods

### Sampling

150 colonies were sampled from nine locations: Cambará do Sul, Herval d’Oeste, Joaçaba, Luzerna, Porto Alegre (PUCRS and UFRGS), Riozinho, Rolante and São Leopoldo. Colonies were located in hives or natural cavities located in tree holes or trunks (Supplementary Table S1). From each colony we collected 15 bees with exception of 20 colonies where the sample size was smaller (Cambará do Sul: *M. bicolor*–1 colony n = 8, *P. remota*–1 colony n = 10 and 1 colony n = 13, *P. saiqui*–1 colony n = 14; Herval d’Oeste: *T. fiebrigi*–1 colony n = 12, *A. mellifera*–1 colony n = 13, *P. emerina*–1 colony n = 14; Luzerna: *P. emerina*–1 colony n = 13; Porto Alegre/PUCRS: *T. fiebrigi*–1 colony n = 13, *P. emerina*–1 colony n = 14 and 1 colony n = 10; Riozinho: *P. emerina*–1 colony n = 13, *M. bicolor*–1 colony n = 14 and 1 colony n = 13; Rolante*: P. emerina*–1 colony n = 13, *M. bicolor*–2 colonies n = 14; São Leopoldo: *T. fiebrigi*–3 colonies n = 14). Bees were stored in 90% ethanol at −20 °C after collection.

### DNA extraction

Before homogenization bees were washed 3 times with ultrapure water to wash of any ethanol remnants. Bees were pooled together per hive and crushed with mortar and pestle with 3 times 500 μl RLT buffer (RNeasy Mini Kit, Qiagen, the Netherlands). The homogenate was collected and vortexed for 1 min. After centrifugation (2 min 1000 g) 30 μl of supernatant was added to 200 μl of InstaGene™ Matrix Solution (Bio-Rad, Hercules, CA, USA). The mixture was incubated for 90 min at 55 °C with shaking (450 rpm) followed by 15 min at 97 °C and shaking (450 rpm). The incubated mixture was then centrifuged for 5 min at 20 817 g and 30 μl of supernatant was collected. The supernatant was centrifuged as before and 25 μl supernatant was taken and stored at −20 °C until further use.

### Pathogen detection

The detection of Nosematidae, Neogregarinorida and Leishmaniinae was done with broad range primers (Supplementary Table S2 and Supplementary Figures S2–S4), all samples were tested for quality by including an internal reference control (primers see Supplementary Table S2). PCR reaction contained 0.5 μM of each primer; 1.5 mM MgCl_2_; 0.4 mM dNTP; 1.25 U Taq DNA polymerase (Invitrogen, Merelbeke, Belgium) and 1 μl sample. PCR reactions were performed in a Sensoquest Lab- cycler using the following protocol: 2 min at 94 °C and 35 amplification cycles (30 s at 94 °C, 30 s at 56 °C, 45 s at 72 °C) and then 3 min at 72 °C. PCR products were analyzed on a 1.5% agarose gel and stained with ethidium bromide visualization was done with a digital camera (Bio-Rad, Hercules, CA, USA) and the Quality One software (Bio-Rad, Hercules, CA, USA). The pathogen identity of positive samples was determined by direct sanger sequencing (LGC, Middlesex, UK). For Leishmaniinae positives, the ITS regions was PCR amplified (same protocol as mentioned above) and send for direct sequencing to identify to species level, as the screening primers did not allow discrimination between certain Trypanosomatid species (primers see Supplementary Table S2).

**Figure 2:**
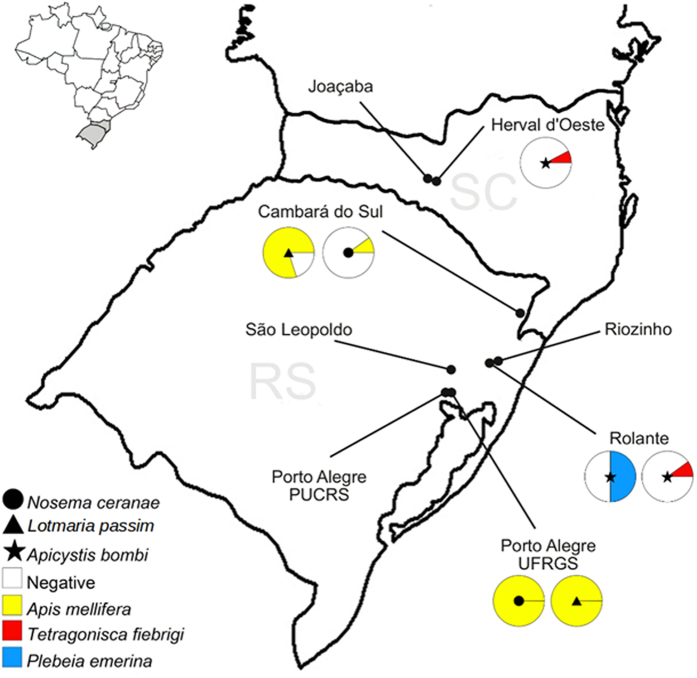
Prevalence of *Lotmaria passim*, *Nosema ceranae* and *Apicystis bombi* in *Apis mellifera* and stingless bees (*Melipona bicolor*, *Plebeia droryana*, *Plebeia emerina*, *Plebeia remota*, *Plebeia saiqui*, *Tetragonisca fiebrigi*) in southern Brazil. Figure modified with Scribus version 1.4.5. (2015)^32^ from http://portaldemapas.ibge.gov.br. Contents extracted from the legislation of the Presidency Portal of the Republic as 21/07/2015, for information purposes only.

The map in Fig. 2 was generated using the open source software Scribus version 1.4.5. (2015) and manual^32^.

### Phylogenetic analysis

The ITS region of *A. bombi* (originating from an infected *Tetragonisca fiebrigi* colony of Rolante) was cloned using the CloneJET PCR Cloning Kit (Life technologies, Gent, Belgium) and isolated with the Plasmid Mini Kit I (Omega Bio-Tek, Norcross, GA, USA). Sequence was obtained by sequencing of plasmids with pJET1.2 primer and were BLAST-searched for confirmation. Sequence was aligned with other ITS sequences from NCBI using ClustalW and subsequently trimmed in MEGA6^33^. Phylogenetic tree was constructed with maximum likelihood using the Hasegawa-Kishino-Yano model.

## Additional Information

**How to cite this article**: Nunes-Silva, P. *et al*. Absence of Leishmaniinae and Nosematidae in stingless bees. *Sci. Rep.* **6**, 32547; doi: 10.1038/srep32547 (2016).

## Supplementary Material

Supplementary Information
