# Supplementary material for: Absence of Leishmaniinae and Nosematidae in stingless bees
Source: Sci Rep. 2016 Sep 2;6:32547. doi: 10.1038/srep32547 (PMC5009319; doi:10.1038/srep32547)
Supplement: Supplementary Information [file srep32547-s1.docx]

**Supplementary data**

**Absence of Leishmaniinae and Nosematidae in stingless bees**

Patrícia Nunes-Silva^1^*, Niels Piot^2^*, Ivan Meeus^2^, Betina Blochtein^1^, Guy Smagghe^2£^

^1^ Laboratory of Entomology, Department of Biodiversity and Ecology, Faculty of Biosciences, Pontifical Catholic University of Rio Grande do Sul, Brazil;

^2^ Laboratory of Agrozoology, Department of Crop Protection, Faculty of Bioscience Engineering, Ghent University, Belgium;

*These authors contributed equally to this work

^£^Corresponding author: guy.smagghe@ugent.be

**Supplementary Table S1**. Detailed sampling table and pathogen incidence in stingless bees and honey bees in nine locations of southern Brazil: 1. Cambará do Sul; 2. Herval d’Oeste; 3. Joaçaba; 4. Luzerna; 5. Porto Alegre – UFRGS (Universidade Federal do Rio Grande do Sul); 6. Porto Alegre – PUCRS (Pontifícia Universidade Católica do Rio Grande do Sul); 7. Riozinho; 8. Rolante; 9. São Leopoldo.

| Location | Species | Nesting substrate | Number of colonies examined | *Apicystis*  *bombi* | *Lotmaria passim* | *Nosema ceranae* |
| --- | --- | --- | --- | --- | --- | --- |
| 1 | *Apis mellifera* | Hive | 10 | 0 | 8 | 1 |
|  | *Melipona bicolor* | Tree | 2 | 0 | 0 | 0 |
|  | *Plebeia emerina* | Hive | 2 | 0 | 0 | 0 |
|  | *Plebeia remota* | Hive | 2 | 0 | 0 | 0 |
|  | *Plebeia saiqui* | Trunk | 3 | 0 | 0 | 0 |
|  |  | Tree | 3 | 0 | 0 | 0 |
| 2 | *A. mellifera* | Hive | 3 | 0 | 0 | 0 |
|  | *P. emerina* | Hive | 3 | 0 | 0 | 0 |
|  |  | Trunk | 1 | 0 | 0 | 0 |
|  |  | Tree | 1 | 0 | 0 | 0 |
|  | *Tetragonisca fiebrigi* | Hive | 10 | 0 | 0 | 0 |
|  |  | Tree | 3 | 1 | 0 | 0 |
| 3 | *T. fiebrigi* | Wall | 2 | 0 | 0 | 0 |
| 4 | *A. mellifera* | Hive | 2 | 0 | 0 | 0 |
|  |  | Tree | 1 | 0 | 0 | 0 |
|  | *P. emerina* | Hive | 3 | 0 | 0 | 0 |
|  |  | Trunk | 1 | 0 | 0 | 0 |
|  |  | Tree | 6 | 0 | 0 | 0 |
|  | *T. fiebrigi* | Tree | 5 | 0 | 0 | 0 |
| 5 | *A. mellifera* | Hive | 4 | 0 | 4 | 4 |
|  | *Plebeia droryana* | Hive | 5 | 0 | 0 | 0 |
|  |  | Tree | 3 | 0 | 0 | 0 |
|  | *T. fiebrigi* | Hive | 3 | 0 | 0 | 0 |
|  |  | Tree | 3 | 0 | 0 | 0 |
|  |  | Wall | 1 | 0 | 0 | 0 |
| 6 | *P. droryana* | Tree | 1 | 0 | 0 | 0 |
|  | *P. emerina* | Tree | 4 | 0 | 0 | 0 |
|  | *T. fiebrigi* | Hive | 8 | 0 | 0 | 0 |
|  |  | Tree | 6 | 0 | 0 | 0 |
|  |  | Wall | 6 | 0 | 0 | 0 |
| 7 | *M. bicolor* | Hive | 2 | 0 | 0 | 0 |
|  | *P. droryana* | Hive | 1 | 0 | 0 | 0 |
|  | *P. emerina* | Hive | 6 | 0 | 0 | 0 |
|  | *P. remota* | Hive | 4 | 0 | 0 | 0 |
|  | *P. saiqui* | Hive | 2 | 0 | 0 | 0 |
|  | *T. fiebrigi* | Hive | 2 | 0 | 0 | 0 |
| 8 | *M. bicolor* | Hive | 6 | 0 | 0 | 0 |
|  | *P. emerina* | Hive | 4 | 2 | 0 | 0 |
|  | *T. fiebrigi* | Hive | 10 | 1 | 0 | 0 |
| 9 | *P. droryana* | Hive | 1 | 0 | 0 | 0 |
|  | *T. fiebrigi* | Hive | 5 | 0 | 0 | 0 |
|  | **Total** |  | 150 | 4 (2.7%) | 12 (8%) | 5 (3.3%) |

**Supplementary Table S2:** Sequences of the primers used for the screening and identification of trypanosomatid species, indicated with ‘*’

| Pathogen | Forward 5’->3’ | Reverse 5’->3’ | Target | Ref. |
| --- | --- | --- | --- | --- |
| Leishmaniinae | CTTTTGGTCGGTGGAGTGAT | GGACGTAATCGGCACAGTTT | 18S rDNA | ^1^ |
| Nosematidae | TATGCCGACGATGTGATATG | CACAGCATCCATTGAAAACG | 18S rDNA | ^2^ |
| *Apicystis* *sp*. | CCAGCATGGAATAACATGTAAGG | GACAGCTTCCAATCTCTAGTCG | 18S rDNA | ^1^ |
| Internal amplification control | AGATGGGGGCATTCGTATTG | ATCTGATCGCCTTCGAACCT | 18S rDNA | ^1^ |
| Trypanosomatid identification* | GCTGTAGGTGAACCTGCAGCAGCTGGATCATT | GGAAGCCAAGTCATCCATC | ITS1 | ^3^ |

**Supplementary Figure S1:** Phylogenetic analysis of *Apicystis bombi* internal transcribed spacers (ITS). The ITS region includes the partial 18S rDNA, complete ITS 1, 5.8S rDNA gene, ITS 2, and the partial 28S rDNA gene. Accession numbers are given as well as the geographical origin and the host. ITS sequence of *Apicystis bombi* originating from stingless bee (*Tetragonisca fiebrigi)* indicated with red dot.


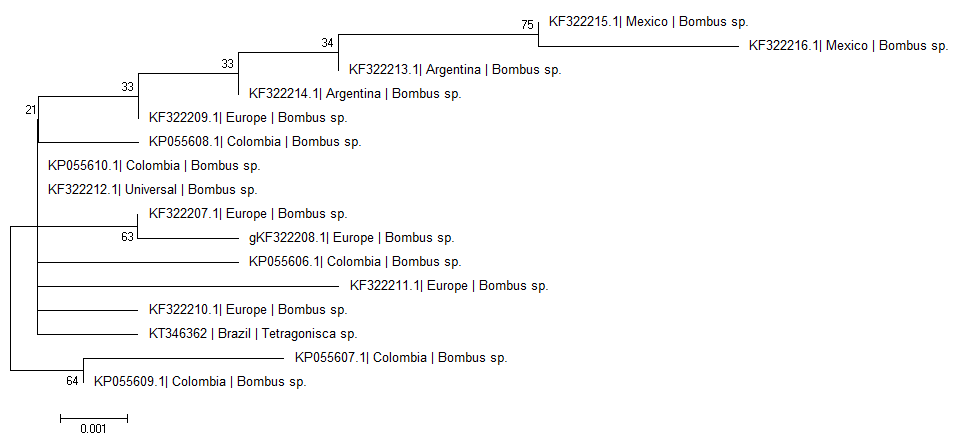


**Supplementary Figure S2:** ClustalW alignment of the 18S rDNA of representative species of each genus of the family of Trypanosomatidae (Trypanosomatina:Kinetoplastida), as well as two species of the Cryptobiidae family (Bodonina:Kinetoplastida); Consensus sequence is indicated with asterix sign; Forward primer as well as reverse primer binding sites are highlighted in green. (Alignment and subsequent trimming was performed in MEGA6^4^ )

Trypanosoma_evansi TGGAGTGTTCTTTCTCGATCCCCTGAATGGTGGTGCATGGCCGCTTTTGGTCGGTGGAGT

Crithidia_deanei TTGAGTGTTCTTTCTCGATTTCTTGAATGGTGGTGCATGGCCGCTTTTGGTCGGTGGAGT

Crithidia_oncopelti TTGAGTGTTCTTTCTCGATTTCTTGAATGGTGGTGCATGGCCGCTTTTGGTCGGTGGAGT

Phytomonas_nordicus TTGAGTGTTCTTTCTCGATTCCCTGAATGGTGGTGCATGGCCGCTTTTGGTCGGTGGAGT

Phytomonas_serpens TTGAGTGTTCTTTCTCGATTCCCTGAATGGTGGTGCATGGCCGCTTTTGGTCGGTGGAGT

Herpetomonas_ztiplika TTGAGTGTTCTTTCTCGATTCCCTGAATGGTGGTGCATGGCCGCTTTTGGTCGGTGGAGT

Herpetomonas_pessoai TTGAGTGTTCTTTCTCGATTCCCTGAATGGTGGTGCATGGCCGCTTTTGGTCGGTGGAGT

Trypanosoma_cruzi_marinkellei TTGAGTGTTCTTTCTCGATCCCCTGAATGGTGGTGCATGGCCGCTTTTGGTCGGTGGAGT

Blastocrithidia_sp. TTGAGTGTTCTTTCTCGATTTCTTGAATGGTGGTGCATGGCCGCTTTTGGTCGGTGGAGT

Crithidia_dedva TTGAGTGTTCTTTCTCGATTCCCTGAATGGTGGTGCATGGCCGCTTTTGGTCGGTGGAGT

Endotrypanum_sp. TTGAGTGTTCTTTCTCGATTCCCTGAATGGTGGTGCATGGCCGCTTTTGGTCGGTGGAGT

Crithidia_otongatchiensis TTGAGTGTTCTTTCTCGATTCCCTGAATGGTGGTGCATGGCCGCTTTTGGTCGGTGGAGT

Crithidia_pragensis TTGAGTGTTCTTTCTCGATTCCCTGAATGGTGGTGCATGGCCGCTTTTGGTCGGTGGAGT

Leptomonas_seymouri TTGAGTGTTCTTTCTCGATTCCCTGAATGGTGGTGCATGGCCGCTTTTGGTCGGTGGAGT

Lotmaria_passim -------------------------------------------CTTTTGGTCGGTGGAGT

Leishmania_donovani TTGAGTGTTCTTTCTCGATTCCCTGAATGGTGGTGCATGGCCGCTTTTGGTCGGTGGAGT

Leishmania_mexicana TTGAGTGTTCTTTCTCGATTCCCTGAATGGTGGTGCATGGCCGCTTTTGGTCGGTGGAGT

Crithidia_bombi -----------------ATT-----------------------CTTTTGGTCGGTGGAGT

Leptomonas_peterhoffi TTGAGTGTTCTTTCTCGATTCCCTGAATGGTGGTGCATGGCCGCTTTTGGTCGGTGGAGT

Crithidia_insperata TTGAGTGTTCTTTCTCGATTCCCTGAATGGTGGTGCATGGCCGCTTTTGGTCGGTGGAGT

Crithidia_confusa TTGAGTGTTCTTTCTCGATTCCCTGAATGGTGGTGCATGGCCGCTTTTGGTCGGTGGAGT

Crithidia_mellificae -------------------------------------------CTTTTGGTCGGTGGAGT

Procryptobia_sorokini TTGATAGTTCTTTCTCGATCCCTTGAATGGTGGTGCATGGCCGCTTTTGGTCGGTGGAGT

Trypanoplasma_borreli TTGAGTGTTCTTTCTCGATCCCTTGAATGGTGGTGCATGGCCGCTTTTGGTCGGTGGAGT

*****************

Trypanosoma_evansi GATTTGTTTGGTTGATTCCGTCAACGGACGAGATCCAAGCTGCCCAGTAGGTGCCGGGAT

Crithidia_deanei GATTTGTTTGGTTGATTCCGTCAACGGACGAGATCCAAGCTGCCCAGTAGAATCCAGAAT

Crithidia_oncopelti GATTTGTTTGGTTGATTCCGCCAACGGACGAGATCCAAGCTGCCCAGTAGAATCCAGAAT

Phytomonas_nordicus GATTTGTTTGGTTGATTCCGTCAACGGACGAGATCCAAGCTGCCCAGTAGAATTCAGAAT

Phytomonas_serpens GATTTGTTTGGTTGATTCCGTCAACGGACGAGATCCAAGCTGCCCAGTAGAATTCAGAAT

Herpetomonas_ztiplika GATTTGTTTGGTTGATTCCGTCAACGGACGAGATCCAAGCTGCCCAGTAGAATTCAGAAT

Herpetomonas_pessoai GATTTGTTTGGTTGATTCCGTCAACGGACGAGATCCAAGCTGCCCAGTAGAATTCAGAAT

Trypanosoma_cruzi_marinkellei GATTTGTTTGGTTGATTCCGTCAACGGACGAGATCCAAGCTGCCCAGTAGGATTCAGAAT

Blastocrithidia_sp. GATTTGTTTGGTTGATTCCGTTAACGGACGAGATCCAAGCTGCCCAGTAGGATTCAGAAT

Crithidia_dedva GATTTGTTTGGTTGATTCCGTCAACGGACGAGATCCAAGCTGCCCAGTAGGATTCAGAAT

Endotrypanum_sp. GATTTGTTTGGTTGATTCCGTCAACGGACGAGATCCAAGCTGCCCAGTAGAATTCAGAAT

Crithidia_otongatchiensis GATTTGTTTGGTTGATTCCGTCAACGGACGAGATCCAAGCTGCCCAGTAGGATTCAGAAT

Crithidia_pragensis GATTTGTTTGGTTGATTCCGTCAACGGACGAGATCCAAGCTGCCCAGTAGAATTCAGAAT

Leptomonas_seymouri GATTTGTTTGGTTGATTCCGTCAACGGACGAGATCCAAGCTGCCCAGTAGAATTCAGAAT

Lotmaria_passim GATTTGTTTGGTTGATTCCGTCAACGGACGAGATCCAAGCTGCCCAGTAGAATTCAGAAT

Leishmania_donovani GATTTGTTTGGTTGATTCCGTCAACGGACGAGATCCAAGCTGCCCAGTAGAATTCAGAAT

Leishmania_mexicana GATTTGTTTGGTTGATTCCGTCAACGGACGAGATCCAAGCTGCCCAGTAGAATTCAGAAT

Crithidia_bombi GATTTGTTTGGTTGATTCCGTCAACGGACGAGATCCAAGCTGCCCAGTAGAATTCAGAAT

Leptomonas_peterhoffi GATTTGTTTGGTTGATTCCGTCAACGGACGAGATCCAAGCTGCCCAGTAGAATTCAGAAT

Crithidia_insperata GATTTGTTTGGTTGATTCCGTCAACGGACGAGATCCAAGCTGCCCAGTAGAATTCAGAAT

Crithidia_confusa GATTTGTTTGGTTGATTCCGTCAACGGACGAGATCCAAGCTGCCCAGTAGAATTCAGAAT

Crithidia_mellificae GATTTGTTTGGTTGATTCCGTCAACGGACGAGATCCAAGCTGCCCAGTAGAATTCAGAAT

Procryptobia_sorokini GATTTGTTTGGTTGATTCCTTTAACGGACGAGATCCAAGCTGCCCAGTAGGAACCAGGAT

Trypanoplasma_borreli GATTTGTTTGGTTGATTCCGTCAACGGACGAGATCCAAGCTGCCCAGTAGGAACCAGGAT

******************* **************************** * * **

Trypanosoma_evansi TGTCCACACAGGACAGCAGTCCCTCCGGCGGGGATTTTTTCCCCAACGGTGGTCGTCATC

Crithidia_deanei TGTC--CATAGGATAGCTTACTCGCTGGCGGC----TTCTTGCCAACGGCGACCGACATT

Crithidia_oncopelti TGTC--CATAGGATAGCTTACTCGCTGGCGGC----TTCTTGCCAACGGCGACCGACATT

Phytomonas_nordicus TATT--CAGAGAATACCAATCTCACTCGCGGG--TCTCTGACCCAACGGTGAGTCGCATT

Phytomonas_serpens TATT--CATAGAATAGCAATCTCACTCGCGGG----TCTGACCCAACGGTGAGTCTCATT

Herpetomonas_ztiplika TGTC--CATAGAATAGCAATCCCGCCGCTGGG----TTCTTCCCGGTGGTGGGCAGCATT

Herpetomonas_pessoai TGTC--CATAGAATAGCAATCTCCTCGCTGGG----TTCTTCTCAGTGAGGAGCAGCATT

Trypanosoma_cruzi_marinkellei TGCC--CATAGGATAGCAATCCCTTCCGCGGG----TTTTACCCAAGGGGGGGCGGTATT

Blastocrithidia_sp. TGCC--CATAGGATAGCAATCTCATCGGCGGG----TTTTACCCAACGGTGAGCAGCATT

Crithidia_dedva TGCC--CATAGGAAAGCAAACTCATCGGCGGG----TTTTACCCAACGGTGGGCCGCATT

Endotrypanum_sp. TGTA--CATAGAATAGCAAACTCATCGGCGGG----TTTTACCCAACGGTGGGCCGCATT

Crithidia_otongatchiensis TGCC--CATAGGAAAGCAAACTCATCGGCGGG----TTTTACCCAACGGTGGGCCGCATT

Crithidia_pragensis TGCC--CATAGGATAGCAAACTCATCGGCGGG----TTTTACCCAACGGTGGGCCGCATT

Leptomonas_seymouri TGTC--CATAGAATAGCAAACTCATCGGCGGG----TTTTACCCAACGGTGGGCCGCATT

Lotmaria_passim TGCC--CATAGGATAGCAAACTCATCGGCGGG----TTTTACCCAACGGCGGGCCGCATT

Leishmania_donovani TGCC--CATAGGATAGCAAACTCATCGGCGGG----TTTTACCCAACGGTGGGCCGCATT

Leishmania_mexicana TGCC--CATAGGATAGCAAACTCATCGGCGGG----TTTTACCCAACGGTGGGCCGCATT

Crithidia_bombi TGCC--CATAGGATAGCAAACTCATCGGCGGG----TTTTACCCAACGGTGGGCCGCATT

Leptomonas_peterhoffi TGCC--CATAGGATAGCAAACTCATCGGCGGG----TTTTACCCAACGGTGGGCCGCATT

Crithidia_insperata TGCC--CATAGGATAGCAAACTCATCGGCGGG----TTTTACCCAACGGTGGGCCGCATT

Crithidia_confusa TGCC--CATAGGATAGCAAACTCATCGGCGGG----TTTTACCCAACGGTGGGCCGCATT

Crithidia_mellificae TGCC--CATAGGATAGCAAACTCATCGGCGGG----TTTTACCCAACGGTGGGCCGCATT

Procryptobia_sorokini TGTC--CAAAAGACAGCAATCTTCTGTGCCGC----TTCGG--CGGCCGGAAGCTACCTC

Trypanoplasma_borreli TGTC--CAAAAGACAGCAATCTTTCGCGTAC-----TTCGGTACAA--GGAAGCTACCTC

* ** * * * * * * * *

Trypanosoma_evansi CTTCTTTTTACAGGCCCCTTCTCTGCGGGATTCCTTGCTTTTCGCGCAAGGTGAGATTTT

Crithidia_deanei CGGTC-------GGATTCTTCTCTGCGGGATTCCCTGTT--TTGCACAGGGTGAAATTTT

Crithidia_oncopelti CGGTC-------GGATTCTTCTCTGCGGGATTCCCTGTT--TTGCACAGGGTGAAATTTT

Phytomonas_nordicus CCGTC-------GAATTCTTCTCTGCGGGATTCCTTGTT--TCGCACAAGGTGAGATTTT

Phytomonas_serpens CGGTC-------GAGTTCTTCTCTGCGGGATTCCTTGTC--TTTAGCAAGGTGAGA-TTT

Herpetomonas_ztiplika CGGTC-------GAATTCTTCTCTGCGGGATTCCTTGTT-TTTGCACAAGGTGAGATTTT

Herpetomonas_pessoai CGGTC-------GAATTCTTCTCTGCGGGATTCCTTGTTTTTTGCACAAGGTGAGATTTT

Trypanosoma_cruzi_marinkellei CGCTT-------GTATCCTTCTCTGCGGGATTCCTTGTT--TTGCGCAAGGTGAGATTTT

Blastocrithidia_sp. CCGTT-------GACTCCTTCTCTGCGGGATTCCTTGTTTTTTGCACAAGGTGAGATTTT

Crithidia_dedva CGGTT-------GAATTCTTCTCTGCGGGATTCCTTTGTATTTGCACAAGGTGAAATTTT

Endotrypanum_sp. CGGTC-------GAATTCTTCTCTGCGGGATTCCTTTGTAATTGCACAAGGTGAAATTTT

Crithidia_otongatchiensis CGGTT-------GAATTCTTCTCTGCGGGATTCCTTTGTATTTGCACAAGGTGAAATTTT

Crithidia_pragensis CGGTC-------GAATTCTTCTCTGCGGGATTCCTTTGTATTTGCACAAGGTGAAATTTT

Leptomonas_seymouri CGGTC-------GAATTCTTCTCTGCGGGATTCCTTTGTATTTGCACAAGGTGAAATTTT

Lotmaria_passim CGGTC-------GAATTCTTCTCTGCGGGATTCCTTTGTATTTGCACAAGGTGAGATTTT

Leishmania_donovani CGGTC-------GAATTCTTCTCTGCGGGATTCCTTTGTAATTGCACAAGGTGAAATTTT

Leishmania_mexicana CGGTC-------GAATTCTTCTCTGCGGGATTCCTTTGTAATTGCACAAGGTGAAATTTT

Crithidia_bombi CGGTC-------GAATTCTTCTCTGCGGGATTCCTTTGTATTTGCACAAGGTGAAATTTT

Leptomonas_peterhoffi CGGTT-------GAATTCTTCTCTGCGGGATTCCTTTGTATTTGCACAAGGTGAAATTTT

Crithidia_insperata CGGTC-------GAATTCTTCTCTGCGGGATTCCTTTGTATTTGCACAAGGTGAAATTTT

Crithidia_confusa CGGTC-------GAATTCTTCTCTGCGGGATTCCTTTGTATTTGCACAAGGTGAAATTTT

Crithidia_mellificae CGGTC-------GAATTCTTCTCTGCGGGATTCCTTTGTATTTGCACAAGGTGAAATTTT

Procryptobia_sorokini CAGTT-------GGTTTCTTCTCTGCAGGATTCCCCGT---TTTCGCGGGGTGAGATTTT

Trypanoplasma_borreli CAGTT-------GGTTTCTTCTCTGCAGGATTCCTTGTC--TCTCGCAAGGTGAGATTTT

* * ********* ******* * * ***** * ***

Trypanosoma_evansi GGGCAACAGCAGGTCTGTGATGCTCCTCAATGTTCTGGGCGACACGCGCACTACAATGTC

Crithidia_deanei GGGCAACAGCAGGTCTGTGATGCTCCTCAATGTTCTGGGCGACACGCGCACTACAATGTC

Crithidia_oncopelti GGGCAACAGCAGGTCTGTGATGCTCCTCAATGTTCTGGGCGACACGCGCACTACAATGTC

Phytomonas_nordicus GGGCAACAGCAGGTCTGTGATGCTCCTCAATGTTCTGGGCGACACGCGCACTACAATGTC

Phytomonas_serpens GGGCAACAGCAGGTCTGTGATGCTCCTCAATGTTCTGGGCGACACGCGCACTACAATGTC

Herpetomonas_ztiplika GGGCAACAGCAGGTCTGTGATGCTCCTCAATGTTCTGGGCGACACGCGCACTACAATGTC

Herpetomonas_pessoai GGGCAACAGCAGGTCTGTGATGCTCCTCAATGTTCTGGGCGACACGCGCACTACAATGTC

Trypanosoma_cruzi_marinkellei GGGCAACAGCAGGTCTGTGATGCTCCTCAATGTTCTGGGCGACACGCGCACTACAATGTC

Blastocrithidia_sp. GGGCAACAGCAGGTCTGTGATGCTCCTCAATGTTCTGGGCGACACGCGCACTACAATGTC

Crithidia_dedva GGGCAACAGCAGGTCTGTGATGCTCCTCAATGTTCTGGGCGACACGCGCACTACAATGTC

Endotrypanum_sp. GGGCAACAGCAGGTCTGTGATGCTCCTCAATGTTCTGGGCGACACGCGCACTACAATGTC

Crithidia_otongatchiensis GGGCAACAGCAGGTCTGTGATGCTCCTCAATGTTCTGGGCGACACGCGCACTACAATGTC

Crithidia_pragensis GGGCAACAGCAGGTCTGTGATGCTCCTCAATGTTCTGGGCGACACGCGCACTACAATGTC

Leptomonas_seymouri GGGCAACAGCAGGTCTGTGATGCTCCTCAATGTTCTGGGCGACACGCGCACTACAATGTC

Lotmaria_passim GGGCAACAGCAGGTCTGTGATGCTCCTCAATGTTCTGGGCGACACGCGCACTACAATGTC

Leishmania_donovani GGGCAACAGCAGGTCTGTGATGCTCCTCAATGTTCTGGGCGACACGCGCACTACAATGTC

Leishmania_mexicana GGGCAACAGCAGGTCTGTGATGCTCCTCAATGTTCTGGGCGACACGCGCACTACAATGTC

Crithidia_bombi GGGCAACAGCAGGTCTGTGATGCTCCTCAATGTTCTGGGCGACACGCGCACTACAATGTC

Leptomonas_peterhoffi GGGCAACAGCAGGTCTGTGATGCTCCTCAATGTTCTGGGCGACACGCGCACTACAATGTC

Crithidia_insperata GGGCAACAGCAGGTCTGTGATGCTCCTCAATGTTCTGGGCGACACGCGCACTACAATGTC

Crithidia_confusa GGGCAACAGCAGGTCTGTGATGCTCCTCAATGTTCTGGGCGACACGCGCACTACAATGTC

Crithidia_mellificae GGGCAACAGCAGGTCTGTGATGCTCCTCAATGTTCTGGGCGACACGCGCACTACAATGTC

Procryptobia_sorokini GGGCAACAGCAGGTCTGTGATGCTCCTCAATGTTCTGGGCGACACGCGCACTACAATGTC

Trypanoplasma_borreli GGGCAACAGCAGGTCTGTGATGCTCCTCAATGTTCTGGGCGACACGCGCACTACAATGTC

************************************************************

Trypanosoma_evansi AGTGAGAACAAGAGTCCGAGCGGCACTTCACAATGTCGCT--CCCGCTTGAT-CAAAAGA

Crithidia_deanei AGTGAGAACAAGA--------AAAACGATATTTCGTCGGA--CCTACATGAT-CAAAAGT

Crithidia_oncopelti AGTGAGAACAAGA--------AAAACGATATTTCGTCGGA--CCTACATGAT-CAAAAGT

Phytomonas_nordicus AGTGAGAACAAGA--------AAAACGACCTA-TGTCGGA--CCTACTTGAT-CAAAAGA

Phytomonas_serpens AGTGAGAACAAGA--------AAAACGACGTA-TGTCGGA--CCTACTTGAT-CAAAAGA

Herpetomonas_ztiplika AGTGAGAACAAGA--------AAAACGACTTA-CGTCGGA--CCTACTTGAT-CAAAAGA

Herpetomonas_pessoai AGTGAGAACAAGA--------AAAACGACTTT-TGTCGGA--CCTACTTGAT-CAAAAGA

Trypanosoma_cruzi_marinkellei AGTGAGAACAAGA--------AAAACGACTCT-TGTCGGA--CCTACTTGAT-CAAAAGA

Blastocrithidia_sp. AGTGAGAACAAGA--------AAAATGACTTT-TGTCAGA--CCTACTTGAT-CAAAAGA

Crithidia_dedva AGTGAGAACAAGA--------AAAACGACTTC-TGTCGAA--CCTACTTGAT-CAAAAGA

Endotrypanum_sp. AGTGAGAACAAGA--------AAAACGACTTT-TGTCGAA--CCTACTTGAT-CAAAAGA

Crithidia_otongatchiensis AGTGAGAACAAGA--------AAAACGACTTT-TGTCGAA--CCTACTTGAT-CAAAAGA

Crithidia_pragensis AGTGAGAACAAGA--------AAAACGACTT--TGTCGAA--CCTACATGAT-CAAAAGA

Leptomonas_seymouri AGTGAGAACAAGA--------AAAACGACTTT-TGTCGAA--CCTACTTGAT-CAAAAGA

Lotmaria_passim AGTGAGAACAAGA--------AAAACGACTTT-TGTCGAA--CCTACTTGAT-CAAAAGA

Leishmania_donovani AGTGAGAACAAGA--------AAAACGACTTT-TGTCGAA--CCTACTTGAT-CAAAAGA

Leishmania_mexicana AGTGAGAACAAGA--------AAAACGACTTT-TGTCGAA--CCTACTTGAT-CAAAAGA

Crithidia_bombi AGTGAGAACAAGA--------AAAACGACTTT-TGTCGAA--CCTACTTGAT-CAAAAGA

Leptomonas_peterhoffi AGTGAGAACAAGA--------AAAACGACTTT-TGTCGAA--CCTACTTGAT-CAAAAGA

Crithidia_insperata AGTGAGAACAAGA--------AAAACGACTTT-TGTCGAA--CCTACTTGAT-CAAAAGA

Crithidia_confusa AGTGAGAACAAGA--------AAAACGACTTT-TGTCGAA--CCTACTTGAT-CAAAAGA

Crithidia_mellificae AGTGAGAACAAGA--------AAAACGACTTT-TGTCGAA--CCTACTTGAT-CAAAAGA

Procryptobia_sorokini AGTGAGAACAAGA--------T-----TCCCT-TACCGGAGTCCCGCTTGATCCAAGAGA

Trypanoplasma_borreli AGTGAGAACAAGA--------T-----TCCCT-TACCGGAGTCCTGCTTGATTCAAAAGA

************* * ** * **** *** **

Trypanosoma_evansi GCGGGGAAACCACGGAATCACGTAGACCCA-CTTGGGACCGAGTATTGCAATTATTGGTC

Crithidia_deanei GTGGGGAATCCACGGAATCACATAGACCCA-CTTGGGACCGAGGATTGCAATTATTGGTC

Crithidia_oncopelti GTGGGGAATCCACGGAATCACATAGACCCA-CTTGGGACCGAGGATTGCAATTATTGGTC

Phytomonas_nordicus GTGGGGAAACCACGGAATCACGTAGACCCA-CTTGGGACCGAGGATTGCAATTATTGGTC

Phytomonas_serpens GTGGGGAAACCACGGAATCACATAGACCCA-CTTGGGACCGAGGATTGCAATTATTGGTC

Herpetomonas_ztiplika GTGGGGAAACCCCGGAATCACATAGACCCGCTTTGGGACCGAGGATTGCAATTATTGGTC

Herpetomonas_pessoai GTGGGGAAACCCCGGAATCACATAGACCCGTTTTGGGACCGAGGATTGCAATTATTGGTC

Trypanosoma_cruzi_marinkellei GTGGGAAAACCCCGGAATCACGTAGACCCA-CTTGGGACCGAGTATTGCAATTATTGGTC

Blastocrithidia_sp. GTGGGGAAACCCCGGAATCACGTAGACCCA-ACTGGGACCGAGGATTGCAATTATTGGTC

Crithidia_dedva GTGGGGAAACCACGGAATCACATAGACCCA-CTTGGGACCGAGGATTGCAATTATTGGTC

Endotrypanum_sp. GTGGGGAAACCCCGGAATCACATAGACCCA-CTTGGGACCGAGGATTGCAATTATTGGTC

Crithidia_otongatchiensis GTGGGGAAACCACGGAATCACATAGACCCA-CTTGGGACCGAGGATTGCAATTATTGGTC

Crithidia_pragensis GTGGGGAAACCCCGGAATCACATAGACCCA-CGCGGGACCGAGGATTGCAATTATTGGTC

Leptomonas_seymouri GTGGGGAAACCCCGGAATCACATAGACCCA-CTTGGGACCGAGGATTGCAATTATTGGTC

Lotmaria_passim GTGGGGAAACCCCGGAATCACATAGACCCA-CTTGGGACCGAGGATTGCAATTATTGGTC

Leishmania_donovani GTGGGGAAACCCCGGAATCACATAGACTCA-CTTGGGACCGAGGATTGCAATTATTGGTC

Leishmania_mexicana GTGGGGAAACCCCGGAATCACATAGACCCA-CTTGGGACCGAGGATTGCAATTATTGGTC

Crithidia_bombi GTGGGGAAACCCAGGAATCACATAGACCCA-CTTGGGACCGAGGATTGCAATTATTGGTC

Leptomonas_peterhoffi GTGGGGAAACCCCGGAATCACATAGACCCA-CTTGGGACCGAGGATTGCAATTATTGGTC

Crithidia_insperata GTGGGGAAACCCCGGAATCACATAGACCCA-CTTGGGACCGAGGATTGCAATTATTGGTC

Crithidia_confusa GTGGGGAAACCCCGGAATCACATAGACCCA-CTTGGGACCGAGGATTGCAATTATTGGTC

Crithidia_mellificae GTGGGGAAACCCCGGAATCACATAGACCCA-CTTGGGACCGAGGATTGCAATTATTGGTC

Procryptobia_sorokini GCGGGGAATCACCGGAATCACATAGACCCG-ATTGGGACCGAGGATTGCAATTATTGGTC

Trypanoplasma_borreli GCGGGGAATCACCGGAATCACATAGACCCA-ATTGGGACCGAGGATTGCAATTATTGGTC

* *** ** * ******** ***** * ********* ****************

Trypanosoma_evansi GCGCAACGAGGAATGTCTCGTAGGCGCAGCTCATCAAACTGTGCCGATTACGTCCCTGCC

Crithidia_deanei GCGCAACGAGGAATGTCTCGTAGGCGCAGCTCATCAAACTGTGCCGATTACGTCCCTGCC

Crithidia_oncopelti GCGCAACGAGGAATGTCTCGTAGGCGCAGCTCATCAAACTGTGCCGATTACGTCCCTGCC

Phytomonas_nordicus GCGCAACGAGGAATGTCTCGTAGGCGCAGCTCATCAAACTGTGCCGATTACGTCCCTGCC

Phytomonas_serpens GCGCAACGAGGAATGTCTCGTAGGCGCAGCTCATCAAACTGTGCCGATTACGTCCCTGCC

Herpetomonas_ztiplika GCGCAACTAGGAATGTCTCGTAGGCGCAGCTCATCAAACTGTGCCGATTACGTCCCTGCC

Herpetomonas_pessoai GCGCAACTAGGAATGTCTCGTAGGCGCAGCTCATCAAACTGTGCCGATTACGTCCCTGCC

Trypanosoma_cruzi_marinkellei GCGCAACGAGGAATGTCTCGTAGGCGCAGCTCATCAAACTGTGCCGATTACGTCCCTGCC

Blastocrithidia_sp. GCGCAACGAGGAATGTCTCGTAGGCGCAGCTCATCAAACTGTGCCGATTACGTCCCTGCC

Crithidia_dedva GCGCAACGAGGAATGTCTCGTAGGCGCAGCTCATCAAACTGTGCCGATTACGTCCCTGCC

Endotrypanum_sp. GCGCAACGAGGAATGTCTCGTAGGCGCAGCTCATCAAACTGTGCCGATTACGTCCCTGCC

Crithidia_otongatchiensis GCGCAACGAGGAATGTCTCGTAGGCGCAGCTCATCAAACTGTGCCGATTACGTCCCTGCC

Crithidia_pragensis GCGCAACGAGGAATGTCTCGTAGGCGCAGCTCATCAAACTGTGCCGATTACGTCCCTGCC

Leptomonas_seymouri GCGCAACGAGGAATGTCTCGTAGGCGCAGCTCATCAAACTGTGCCGATTACGTCCCTGCC

Lotmaria_passim GCGCAACGAGGAATGTCTCGTAGGCGCAGCTCATCAAACTGTGCCGATTACGTCC-----

Leishmania_donovani GCGCAACGAGGAATGTCTCGTAGGCGCAGCTCATCAAACTGTGCCGATTACGTCCCTGCC

Leishmania_mexicana GCGCAACGAGGAATGTCTCGTAGGCGCAGCTCATCAAACTGTGCCGATTACGTCCCTGCC

Crithidia_bombi GCGCAACGAGGAATGTCTCGTAGGCGCAGCTCATCAAACTGTGCCGATTACGTCC-----

Leptomonas_peterhoffi GCGCAACGAGGAATGTCTCGTAGGCGCAGCTCATCAAACTGTGCCGATTACGTCCCTGCC

Crithidia_insperata GCGCAACGAGGAATGTCTCGTAGGCGCAGCTCATCAAACTGTGCCGATTACGTCCCTGCC

Crithidia_confusa GCGCAACGAGGAATGTCTCGTAGGCGCAGCTCATCAAACTGTGCCGATTACGTCCCTGCC

Crithidia_mellificae GCGCAACGAGGAATGTCTCGTAGGCGCAGCTCATCAAACTGTGCCGATTACGTCC-----

Procryptobia_sorokini GCGCAACGAGGAATGTCTCGTAGGCGCAGCTCATCAAACTGTGCCGATTACGTCCCTGCC

Trypanoplasma_borreli GCGCAACGAGGAATGTCTCGTAGGCGCAGTTCATCAAACTGTGCCGATTACGTCCCTGCC

******* ********************* *************************

| **Species** | **Family** | **Suborder** | **Order** | **Accession** |
| --- | --- | --- | --- | --- |
| *Trypanosoma evansi* | Trypanosomatidae | Trypanosomatina | Kinetoplastida | AY912269.1 |
| *Crithidia deanei* | Trypanosomatidae | Trypanosomatina | Kinetoplastida | EU099538.1 |
| *Crithidia oncopelti* | Trypanosomatidae | Trypanosomatina | Kinetoplastida | EU099539.1 |
| *Phytomonas nordicus* | Trypanosomatidae | Trypanosomatina | Kinetoplastida | KT223609.1 |
| *Phytomonas serpens* | Trypanosomatidae | Trypanosomatina | Kinetoplastida | AF016323.1 |
| *Herpetomonas ztiplika* | Trypanosomatidae | Trypanosomatina | Kinetoplastida | AF416560.2 |
| *Herpetomonas pessoai* | Trypanosomatidae | Trypanosomatina | Kinetoplastida | JQ359718.1 |
| *Trypanosoma cruzi* | Trypanosomatidae | Trypanosomatina | Kinetoplastida | FJ649484.1\| |
| *Blastocrithidia* sp. | Trypanosomatidae | Trypanosomatina | Kinetoplastida | FJ916992.1 |
| *Crithidia dedva* | Trypanosomatidae | Trypanosomatina | Kinetoplastida | JN624299.1 |
| *Endotrypanum* sp. | Trypanosomatidae | Trypanosomatina | Kinetoplastida | EU021240.1 |
| *Crithidia otongatchiensis* | Trypanosomatidae | Trypanosomatina | Kinetoplastida | JQ658813.1 |
| *Crithidia pragensis* | Trypanosomatidae | Trypanosomatina | Kinetoplastida | KC205988.1 |
| *Leptomonas seymouri* | Trypanosomatidae | Trypanosomatina | Kinetoplastida | AF153040.2 |
| *Lotmaria passim* | Trypanosomatidae | Trypanosomatina | Kinetoplastida | KJ704237.1 |
| *Leishmania donovani* | Trypanosomatidae | Trypanosomatina | Kinetoplastida | GQ332356.1 |
| *Leishmania mexicana* | Trypanosomatidae | Trypanosomatina | Kinetoplastida | GQ332360.1 |
| *Crithidia bombi* | Trypanosomatidae | Trypanosomatina | Kinetoplastida | KF607065.1 |
| *Leptomonas peterhoffi* | Trypanosomatidae | Trypanosomatina | Kinetoplastida | AF153039 |
| *Crithidia insperata* | Trypanosomatidae | Trypanosomatina | Kinetoplastida | JF717836.1 |
| *Crithidia confusa* | Trypanosomatidae | Trypanosomatina | Kinetoplastida | JF717837.1 |
| *Crithidia mellificae* | Trypanosomatidae | Trypanosomatina | Kinetoplastida | KJ704242.1 |
| *Protocryptobia sorokini* | Cryptobiidae | Bodonina | Kinetoplastida | KF479401.1 |
| *Trypanoplasma borreli* | Cryptobiidae | Bodonina | Kinetoplastida | L14840.1 |

**Supplementary Figure S3:** Clustal-W alignment of the 18S rDNA of known Hymenoptera infecting Nosematidae, with available sequences on NCBI as well as several Lepidoptera and Coleoptera infecting species of the genus *Nosema* and *Vairimorpha*. Consensus sequence is indicated with asterix sign; Forward primer as well as reverse primer binding sites are highlighted in green. (Alignment and subsequent trimming was performed in MEGA6^4^ )

Nosema_bombi GTATGTATTTTTTGAACAAGGACGTAAGCTGGAGGATCGAAGATGATTAGATACCATTGT

Nosema_oulemae GTATGTATTTTTTGAACAAGGACGTAAGCTGGAGGAGCGAAGATGATTAGATACCATTGT

Nosema_necatrix_ATCC GTATGTATTTTTTGAACAAGGACGTAAGCTGGAGGAGCGAAGATGATTAGATACCATTGT

Vairimorpha_necatrix GTATGTATTTTTTGAACAAGGACGTAAGCTGGAGGAGCGAAGATGATTAGATACCATTGT

Nosema_apis GTATGTATTTTTTGAACAAGGACGTAAGCTGGAGGATCGAAGATGATTAGATACCATTGT

Nosema_ceranae GTATGTATTTTTTGAACAAGGACGTAAGCTGGAGGAGCGAAGATGATTAGATACCATTGT

Nosema_sp._KU-9 GTATGTATTTTTTGAACAAGGACGTAAGCTGGAGGAGCGAAGATGATTAGATACCATTGT

Vairimorpha_lymantriae GTATGTATTTTTTGAACAAGGACGTAAGCTGGAGGAGCGAAGATGATTAGATACCATTGT

**Nosema_vespula** GTATGTATTTTTTGAACAAGGACGTAAGCTGGAGGAGCGAAGATGATTAGATACCATTGT

Nosema_portugal GTATGTATTTTTTGAACAAGGACGTAAGCTGGAGGAGCGAAGATGATTAGATACCATTGT

Nosema_chrysorrhoeae GTATGTATTTTTTGAACAAGGACGTAAGCTGGAGGAGCGAAGATGATTAGATACCATTGT

Nosema_thomsoni GTATGTATTTTTTGAACAAGGACGTAAGCTGGAGGAGCGAAGATGATTAGATACCATTGT

Nosema_sp.CHW-2007a GTATGTATTTTTTGAACAAGGACGTAAGCTGGAGGAGCGAAGATGATTAGATACCATTGT

************************************ ***********************

Nosema_bombi AGTTCCAGCAGTAAACTATGCCGACGATGTGATATGA------GTATTTGTATTACATAG

Nosema_oulemae AGTTCCAGCAGTAAACTATGCCGACGATGTGATATGATA----TTTATTGTATTACATGA

Nosema_necatrix_ATCC AGTTCCAGCAGTAAACTATGCCGACGATGTGATATGATA----TTAATTGTATTAGATGA

Vairimorpha_necatrix AGTTCCAGCAGTAAACTATGCCGACGATGTGATATGATA----TTAATTGTATTAGATGA

Nosema_apis AGTTCCAGCAGTAAACTATGCCGACGATGTGATATGA------GATGTTGTATTACATTA

Nosema_ceranae AGTTCCAGCAGTAAACTATGCCGACGATGTGATATGAAAATGTTAATTTGTATTACATAA

Nosema_sp._KU-9 AGTTCCAGCAGTAAACTATGCCGACGATGTGATATGATA----TATTTTGTATTACATAA

Vairimorpha_lymantriae AGTTCCAGCAGTAAACTATGCCGACGATGTGATATGATA----TATTTTGTATTACATAA

Nosema_vespula AGTTCCAGCAGTAAACTATGCCGACGATGTGATATGATA----TATTTTGTATTACATAA

Nosema_portugal AGTTCCAGCAGTAAACTATGCCGACGATGTGATATGATA----TATTTTGTATTACATAA

Nosema_chrysorrhoeae AGTTCCAGCAGTAAACTATGCCGACGATGTGATATGATA----TATTTTGTATTACATAA

Nosema_thomsoni AGTTCCAGCAGTAAACTATGCCGACGATGTGATATGA-A----TATTTTGTATTACATAA

Nosema_sp. CHW-2007a AGTTCCAGCAGTAAACTATGCCGACGATGTGATATGA-A----TATTTTGTATTACATAA

************************************* * ****** **

Nosema_bombi TAGAAATTAGAGTTTTTTGGCTCTGGGGATAGTATGATCGCAAGATTGAAAATTAAAGAA

Nosema_oulemae TAGAAATTTGAGTTTTTTGGCTCTGGGGATAGTATGATCGCAAGATTGAAAATTAAAGAA

Nosema_necatrix_ATCC TAGAAATTTGAGTTTTTTGGCTCTGGGGATAGTATGATCGCAAGATTGAAAATTAAAGAA

Vairimorpha_necatrix TAGAAATTTGAGTTTTTTGGCTCTGGGGATAGTATGATCGCAAGATTGAAAATTAAAGAA

Nosema_apis TAGAAATTAGAGTTTTTTGGCTCTGGGGATAGTATGATCGCAAGATTGAAAATTAAAGAA

Nosema_ceranae TAGAAATTTGAGTTTTTTGGCTCTGGGGATAGTATGATCGCAAGATTGAAAATTAAAGAA

Nosema_sp._KU-9 TAGAAATTAGAGTTTTTTGGCTCTGGGGATAGTATGATCGCAAGATTGAAAATTAAAGAA

Vairimorpha_lymantriae TAGAAATTAGAGTTTTTTGGCTCTGGGGATAGTATGATCGCAAGATTGAAAATTAAAGAA

Nosema_vespula TAGAAATTAGAGTTTTTTGGCTCTGGGGATAGTATGATCGCAAGATTGAAAATTAAAGAA

Nosema_portugal TAGAAATTAGAGTTTTTTGGCTCTGGGGATAGTATGATCGCAAGATTGAAAATTAAAGAA

Nosema_chrysorrhoeae TAGAAATTAGAGTTTTTTGGCTCTGGGGATAGTATGATCGCAAGATTGAAAATTAAAGAA

Nosema_thomsoni TAGAAATTAGAGTTTTTTGGCTCTGGGGATAGTATGATCGCAAGATTGAAAATTAAAGAA

Nosema_sp. CHW-2007a TAGAAACTAGAGTTTTTTGGCTCTGGGGATAGTATGATCGCAAGATTGAAAATTAAAGAA

****** * ***************************************************

Nosema_bombi ATTGACGGAAGAATACCACAAGGAGTGGATTGTGCGGCTTAATTTGACTCAACGCGAGGT

Nosema_oulemae ATTGACGGAAGAATACCACAAGGAGTGGATTGTGCGGCTTAATTTGACTCAACGCGAGGT

Nosema_necatrix_ATCC ATTGACGGAAGAATACCACAAGGAGTGGATTGTGCGGCTTAATTTGACTCAACGCGAGGT

Vairimorpha_necatrix ATTGACGGAAGAATACCAGAAGGAGTGGATTGTGCGGCTTAATTTGACTCAACGCGAGGT

Nosema_apis ATTGACGGAAGAATACCACAAGGAGTGGATTGTGCGGCTTAATTTGACTCAACGCGAGGT

Nosema_ceranae ATTGACGGAAGAATACCACAAGGAGTGGATTGTGCGGCTTAATTTGACTCAACGCGAGGT

Nosema_sp._KU-9 ATTGACGGAAGAATACCACAAGGAGTGGATTGTGCGGCTTAATTTGACTCAACGCGAGGT

Vairimorpha_lymantriae ATTGACGGAAGAATACCACAAGGAGTGGATTGTGCGGCTTAATTTGACTCAACGCGAGGT

Nosema_vespula ATTGACGGAAGAATACCACAAGGAGTGGATTGTGCGGCTTAATTTGACTCAACGCGAGGT

Nosema_portugal ATTGACGGAAGAATACCACAAGGAGTGGATTGTGCGGCTTAATTTGACTCAACGCGAGGT

Nosema_chrysorrhoeae ATTGACGGAAGAATACCACAAGGAGTGGATTGTGCGGCTTAATTTGACTCAACGCGAGGT

Nosema_thomsoni ATTGACGGAAGAATACCACAAGGAGTGGATTGTGCGGCTTAATTTGACTCAACGCGAGGT

Nosema_sp. CHW-2007a ATTGACGGAAGAATACCACAAGGAGTGGATTGTGCGGCTTAATTTGACTCAACGCGAGGT

****************** *****************************************

Nosema_bombi AACTTACCAATATTTTATTATTCTGAGAGAA-----TCCTGATTCGAGAATGATAATAGT

Nosema_oulemae AACTTACCAATATTTTATTATTCAGAGAGAA----TTTTTAATTTGAGGATGATAATAGT

Nosema_necatrix_ATCC AACTTACCAATATTTTATTATTCAGAGAAGA----TTTATAATCTGAGAATGATAATAGT

Vairimorpha_necatrix AACTTACCAATATTTTA---TTCAGAGAAGA----TTTTCGATCTGAGAATGATAATAGT

Nosema_apis AACTTACCAATATTTTATTGTTCTGCGAGGA------TATGATCTGAGGATGATAATAGT

Nosema_ceranae AACTTACCAATATTTTATTATTTTGAGAGAACCGGTTTTTTGTTTGAGAATGATAATAGT

Nosema_sp._KU-9 AACTTACCAATATTTTATTATTTTGAGACGA----TTTTTTATCAGAGAATGATAATAGT

Vairimorpha_lymantriae AACTTACCAATATTTTATTATTTTGAGACGA----TTCTTAATCAGAGAATGATAATAGT

Nosema_vespula AACTTACCAATATTTTATTATTTTGAGACGA----TTTTTAATCAGAGAATGATAATAGT

Nosema_portugal AACTTACCAATATTTTATTATTTTGAGACGA----TTTTTAATCAGAGAATGATAATAGT

Nosema_chrysorrhoeae AACTTACCAATATTTTATTATTTTGAGACGA----TTTTTAATCAGAGAATGATAATAGT

Nosema_thomsoni AACTTACCAATATTTTATTATTTTGAGAGGA----TTTTTAATCTGAGAATGATAATAGT

Nosema_sp. CHW-2007a AACTTACCAATATTTTATTATTTTGAGAGGA----TTTTTAATCTGAGAATGATAATAGT

***************** ** * ** * * *** ***********

Nosema_bombi GGTGCATGGCCGTTTTCAATGGATGCTGTGAAGTTT

Nosema_oulemae GGTGCATGGCCGTTTTCAATGGATGCTGTGAAGTTT

Nosema_necatrix_ATCC GGTGCATGGCCGTTTTCAATGGATGCTGTGAAGTTT

Vairimorpha_necatrix GGTGCATGGCCGTTTTCAATGGATGCTGTGAAGTTT

Nosema_apis GGTGCATGGCCGTTTTCAATGGATGCTGTGAAGTTT

Nosema_ceranae GGTGCATGGCCGTTTTCAATGGATGCTGTGAAGTTT

Nosema_sp._KU-9 GGTGCATGGCCGTTTTCAATGGATGCTGTGAAGTTT

Vairimorpha_lymantriae GGTGCATGGCCGTTTTCAATGGATGCTGTGAAGTTT

Nosema_vespula GGTGCATGGCCGTTTTCAATGGATGCTGTGAAGTTT

Nosema_portugal GGTGCATGGCCGTTTTCAATGGATGCTGTGAAGTTT

Nosema_chrysorrhoeae GGTGCATGGCCGTTTTCAATGGATGCTGTGAAGTTT

Nosema_thomsoni GGTGCATGGCCGTTTTCAATGGATGCTGTGAAGTTT

Nosema_sp. CHW-2007a GGTGCATGGCCGTTTTCAATGGATGCTGTGAAGTTT

************************************

| **Species** | **Family** | **Phylum** | **Host** | **Accession** |
| --- | --- | --- | --- | --- |
| *Nosema bombi* | Nosematidae | Microsporidia | Hymenoptera: Apoidae | AY741104.1 |
| *Nosema oulemae* | Nosematidae | Microsporidia | Coleoptera | U27359.1 |
| *Nosema necatrix ATCC* | Nosematidae | Microsporidia | Lepidoptera | U11051.1 |
| *Vairimorpha necatrix* | Burenellidae | Microsporidia | Lepidoptera | Y00266.1 |
| *Nosema apis* | Nosematidae | Microsporidia | Hymenoptera: Apoidae | FJ789798.1 |
| *Nosema ceranae* | Nosematidae | Microsporidia | Hymenoptera: Apoidae | KC680656.1 |
| *Nosema* sp. *KU-9* | Nosematidae | Microsporidia | Lepidoptera | AF141130.1 |
| *Vairimorpha lymantriae* | Burenellidae | Microsporidia | Lepidoptera | AF033315.1 |
| *Nosema vespula* | Nosematidae | Microsporidia | Hymenoptera | U11047.1 |
| *Nosema portugal* | Nosematidae | Microsporidia | Lepidoptera | AF033316.1 |
| *Nosema chrysorrhoeae* | Nosematidae | Microsporidia | Lepidoptera | AY940656.1 |
| *Nosema thomsoni* | Nosematidae | Microsporidia | Hymenoptera: Apoidae | AB860144.1 |
| *Nosema* sp. CHW-2007a | Nosematidae | Microsporidia | Hymenoptera: Apidae | EF585399.1 |

**Supplementary Figure S4:** Clustal-W alignment of the 18S rDNA of all known Hymenoptera infecting Neogregarinorida, with available sequences on NCBI. Consensus sequence is indicated with asterix sign; Forward primer as well as reverse primer binding sites are highlighted in green. (Alignment and subsequent trimming was performed in MEGA6^4^ )

Apicystis_bombi ACAATTCTCTTAATATTTTATTTTAGCTT---GCTAAAATAAAATGTT--AGAGGTGTTA

Mattesia_geminata GCAATTCTCTGGTCAA---ATCTTTGCTTTACGGCA------AAGGTTTCAGAGGTGTTA

Mattesia_sp. GCAATTCTCTGATAAACTTTTTGTTGCTTTAGGGTAGCAAAGAGTGGTGCAGAGGTGTTA

********* * * * **** * * * * * **********

Apicystis_bombi CTTTGAGAAAATTAGAGTGCTTCAAGCAGGCGTACTGCCCTGAATACTCCAGCATGGAAT

Mattesia_geminata CTTTGAGAAAATTAGAGTGCTTCAAGCAGGCGTTTCGCCCTGAATACTCCAGCATGGAAT

Mattesia_sp. CTTTGAGAAAATTAGAGTGCTTCAAGCAGGCGTTACGCCCTGAATACTCCAGCATGGAAT

********************************* ************************

Apicystis_bombi AACATGTAAGGACTATGGTTCTTCTTGTTGGTTTAAGGACCAAGTAATGATTAATAGGGA

Mattesia_geminata AACATGTAAGGACTATGGTTCTTCTTGTCGGTTTAAGGACCAAGTAATGATTAATAGGGA

Mattesia_sp. AACATGTAAGGACTATGGTTCTTCTTGTCGGTTTAAGGACCAAGTAATGATTAATAGGGA

**************************** *******************************

Apicystis_bombi CAGTTGGGGGCATTCGTATTTAGTAGCTAGAGGTGAAATTCTTAGACCTACTAAAGACGA

Mattesia_geminata CAGTTGGGGGCATTCGTATTTAGTAGCTAGAGGTGAAATTCTTAGACCTACTAAAGACGA

Mattesia_sp. CAGTTGGGGGCATTCGTATTTAGTAGCTAGAGGTGAAATTCTTAGACCTACTAAAGACGA

************************************************************

Apicystis_bombi ACTACTGCGAAAGCATCTGCCAAGGATGTTTTCATTAATCAAGAACGAAAGTTAAGGGAT

Mattesia_geminata ACTACTGCGAAAGCATCTGCCAAGGATGTTTTCATTAATCAAGAACGAAAGTTAAGGGAT

Mattesia_sp. ACTACTGCGAAAGCATCTGCCAAGGATGTTTTCATTAATCAAGAACGAAAGTTAAGGGAT

************************************************************

Apicystis_bombi CGAAGACGATCAGATACCGTCGTAGTCTTAACCATAAACGATGCCGACTAGAGATTGGAA

Mattesia_geminata CGAAGACGATCAGATACCGTCGTAGTCTTAACCATAAACGATGCCGACTAGAGATTGGAA

Mattesia_sp. CGAAGACGATCAGATACCGTCGTAGTCTTAACCATAAACGATGCCGACTAGAGATTGGAA

************************************************************

Apicystis_bombi GCTGTCATGTATACGCCGCTTTCAGCACCTTAAGAGAAATCAAAGTCTTTGGGTTCTGGG

Mattesia_geminata GCTGTCAAAAATACGCCACTTTCAGCACCTTAAGAGAAATCAAAGTCTTTGGGTTCTGGG

Mattesia_sp. GCTGTCAAAAATACGCCACTTTCAGCACCTTAAGAGAAATTAAAGTCTTTGGGTTCTGGG

******* ******* ********************** *******************

| **Species** | **Family** | **Order** | **Host** | **Accession** |
| --- | --- | --- | --- | --- |
| *Apicystis bombi* | Ophryocystidae | Neogregarinorida | Hymenoptera | FN546182.1 |
| *Mattesia geminata* | Lipotrophidae | Neogregarinorida | Hymenoptera | AY334568.1 |
| *Mattesia* sp. | Lipotrophidae | Neogregarinorida | Hymenoptera | AY334569.1 |

**References**

1. Meeus, I., de Graaf, D. C., Jans, K. & Smagghe, G. Multiplex PCR detection of slowly-evolving trypanosomatids and neogregarines in bumblebees using broad-range primers. *J. Appl. Microbiol.* **109,** 107–15 (2010).

2. Ravoet, J. *et al.* Widespread occurrence of honey bee pathogens in solitary bees. *J. Invertebr. Pathol.* **122,** 55–58 (2014).

3. Maia Da Silva, F. *et al.* Phylogeny, taxonomy and grouping of *Trypanosoma rangeli* isolates from man, triatomines and sylvatic mammals from widespread geographical origin based on SSU and ITS ribosomal sequences. *Parasitology* **129,** 549–561 (2004).

4. Tamura, K., Stecher, G., Peterson, D., Filipski, A. & Kumar, S. MEGA6: Molecular Evolutionary Genetics Analysis Version 6.0. *Mol. Biol. Evol.* **30,** 2725–2729 (2013).
